# Clinical Validation Results of an Innovative Non-Invasive Device for Colorectal Cancer Preventive Screening through Fecal Exhalation Analysis

**DOI:** 10.3390/cancers12061471

**Published:** 2020-06-04

**Authors:** Giulia Zonta, Cesare Malagù, Sandro Gherardi, Alessio Giberti, Alessandro Pezzoli, Aldo De Togni, Caterina Palmonari

**Affiliations:** 1 Department of Physics and Earth Sciences, University of Ferrara, Via Savonarola, 9-44121 Ferrara, Italy; malagu@fe.infn.it (C.M.); c.palmonari@ausl.fe.it (C.P.); 2 SCENT (Semiconductor-Based Electronic Network for Tumors) S.r.l., Via Quadrifoglio 11, 44124 Ferrara, Italy; gherardi@fe.infn.it; 3 MIST E-R s.c.r.l. (MISTER Smart Innovation), Via P. Gobetti 101, 40129 Bologna, Italy; giberti@fe.infn.it; 4 St. Anna Hospital, Via Aldo Moro, 44124 Ferrara, Italy; alessandro.pezzoli@unife.it; 5 Department of Public Health (AUSL)—UO Igiene Pubblica—Via Fausto Beretta, 7-44121 Ferrara, Italy; a.detogni@ausl.fe.it

**Keywords:** CRC, colorectal cancer, sensors, preventive screening, clinical validation, biomarkers

## Abstract

Screening is recommended to reduce both incidence and mortality of colorectal cancer. Currently, many countries employ fecal occult blood test (FOBT). In Emilia-Romagna (Italy), since 2005, FOBT immunochemical version (FIT) is performed every two years on people aged between 50 and 69 years. A colonoscopy is then carried out on those who are FIT positive. However, FIT shows approximately 65% false positives (non-tumoral bleedings), leading to many negative colonoscopies. The use of an economic and easy-to-use method to check FOBT-positives will improve screening effectiveness, reducing costs to the national health service. This work illustrates the results of a three-year clinical validation protocol (started in 2016) of a patented device composed of a core of nanostructured gas sensors. This device was designed to identify CRC presence by fecal volatile compounds, with a non-invasive, in vitro and low-cost analysis. Feces are, in fact, affected by tumor-volatile biomarkers, produced by cellular peroxidation and metabolic alterations. The protocol consisted in the analysis of fecal samples of FIT-positive subjects, using colonoscopy as a gold standard. A total of 398 samples were analyzed with machine learning techniques, leading to a sensitivity and specificity of 84.1% and 82.4%, respectively, and a positive predictive value of 72% (25–35% for FIT).

## 1. Introduction

Cancer is the leading cause of death in Western countries. Colorectal cancer (CRC) has the highest incidence (in both sexes) and the second highest mortality after lung cancer [1]. In Europe, in 2015, 154,000 people died from CRC, representing 11.7% of all cancer deaths and 3.0% of total deaths. Moreover, the share of deaths attributed to CRC was 3.3 % for men and 2.6% for women. [2]. Nonetheless, if promptly diagnosed, CRC is also one of the most curable cancers (approx. 90% at stage I) and prevention is fundamental for avoiding further advance [3].

CRC screening programs aim to reduce mortality by detecting advanced adenomas or early stage (I or II) colorectal cancer. Screening for CRC appears to be cost effective compared to no screening. In 2003, based on compelling evidence, the Council of the European Union recommended that all member states should establish early detection programs with CRC screening for men and women aged 50 to 74 years, with annual or biennial FOBTs, followed by colonoscopy when results were positive.

The effectiveness of an organized screening program on the population is key to identifying the presence of CRC before its degeneration, particularly in asymptomatic people. However, as one way of encouraging the population to undergo screening, one aspect to consider is non-invasiveness (i.e., acceptability). Currently, two different types of FOBT are available: the fecal occult blood immuno-test (FIT) that uses antibodies to detect human hemoglobin protein in stool, and the guaiac fecal occult blood test (G-FOBT), in which guaiac—a plant-based substance—is used to coat the FOBT test cards. The FIT achieved significantly higher detection rates for advanced adenomas and CRC than the G-FOBT and, being non-invasive and easy to perform, is currently employed in many states. Moreover, FIT does not require dietary restrictions, due to its specificity to human hemoglobin [Hb], and only one sample is needed in most screening programs. As an example, in the Emilia-Romagna region, as in many other regions of Italy, the Department of Public Health invites all individuals aged between 50 and 69 years to undergo a FIT every two years. All FIT positives are subsequently invited to undergo colonoscopy to further evaluate their health status [4]. FIT is recommended only as a preventive screening tool and not as a diagnostic test. A percentage in the range 26–34% of FOBTs are inappropriately carried out, being performed outside the screening program [5]. In Ferrara, the city in which this work was carried out, FIT has been part of the screening program since 2005 [6]. As a result, many tumors have been identified, notably during the first round of screening (prevalence round) [7]. Despite this important result, there are many side effects, due to the presence of about 65% false positives, inasmuch that the presence of blood in the stool can be due to numerous non-tumoral diseases (e.g., inflammatory diseases, diverticula, hemorrhoids, fissures) [8]. This high number of false positives, calculated on the basis of the data resulting from this protocol, leads to a similarly high number of non-operative colonoscopies performed on patients who do not have a tumor, often elderly and already debilitated. This fact generates a non-negligible risk of complications, such as intestinal perforation [9]. The adoption of non-invasive methods to reduce the number of unnecessary colonoscopies, preserving or even improving the sensitivity of current screening, could mark a turning point for the national health service (NHS), as it would allow access to colonoscopy only to patients with CRC, reducing both screening costs and risk to the patient’s health. By considering these FIT limitations, in preliminary researches of our group we tried to find an alternative method to non-invasively identify cancer in the human body by means of an artificial intestine and identification of tumor biomarkers in flatus. This study began in 2013 at the Sensor Laboratory (SL) of the Department of Physics and Earth Science at the University of Ferrara (UNIFE) by studying various CRC biomarkers mentioned in the literature (e.g., decanal, 1-iodononane, and benzene as representative of benzene-ring compounds) [10,11] to identify the most suitable gas sensor materials capable of recognizing CRC biomarkers in a mixture of interferers commonly present in the intestine as digestive products [12]. To carry out these tests, an artificial intestine was created in the laboratory by means of a hermetically sealed chamber and a pneumatic system [13,14,15]. However, this a priori approach is not suitable to perform on subjects, though it has been useful in setting a baseline for progressing the study towards a new a posteriori approach, in which unknown tumor biomarkers may be analyzed through their total effect.

In this study we present the clinical validation results of a device based on semiconductor gas sensors, named SCENT A1 [16]. The prototype is designed to detect the presence of colorectal adenomas and carcinomas from fecal odor. The difference between fecal samples from healthy and CRC-affected people is due to the presence of volatile biomarkers produced by neoplastic and pre-neoplastic cells in the intestine (e.g., peroxidation products or metabolic alterations) [10,11,17,18,19,20,21,22].

The test with SCENT A1 is a completely non-invasive, low cost, in vitro, and easy to perform.

Once sensors had been selected, they were installed in the first prototype of SCENT A1 for a feasibility study carried out with the St. Anna Hospital in Ferrara (Italy) to test them directly on real fecal samples. Feces from CRC-affected patients, extracted during surgery, were compared with healthy volunteer stools, producing encouraging results (95% specificity and 95% sensitivity) [23,24].

Subsequent to the prototype study, a clinical validation Protocol began, in May 2016, involving St. Anna Hospital in Ferrara, the University of Ferrara (UNIFE), AUSL of Ferrara and the start-up SCENT S.r.l, concluding in July 2019. In this paper, the details, results and future aim of this Protocol are discussed in order to focus on the health (and social) benefits that SCENT A1 test can bring.

## 2. Materials and Methods

### 2.1. Statement of Significance

We present here the results of a three-year clinical trial, validated by Ethics Committee (protocol n: 151298), of a device based on nanostructured sensors on a statistically homogeneous population of 1000. The working principle is based on the detection of fecal volatile organic compounds instead of hemoglobin. Thanks to a significantly higher positive predictive value on colonoscopy (gold standard), the method is capable of increasing the operative colonoscopies with respect to redundant ones.

### 2.2. The SCENT A1 Device and its Outcomes

**Figure 1:**
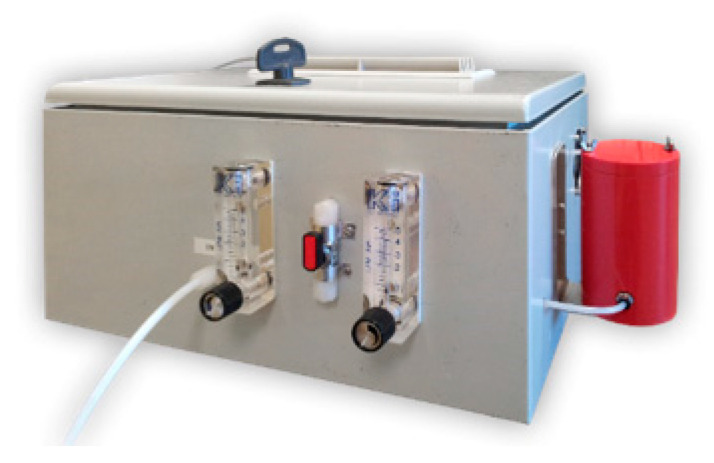
Device SCENT A1. External view.

SCENT A1 (Figure 1) is a portable device composed of a core of five semiconductor gas sensors, a pneumatic system, specific electronics and a sensing unit [16]. 

**Figure 2:**
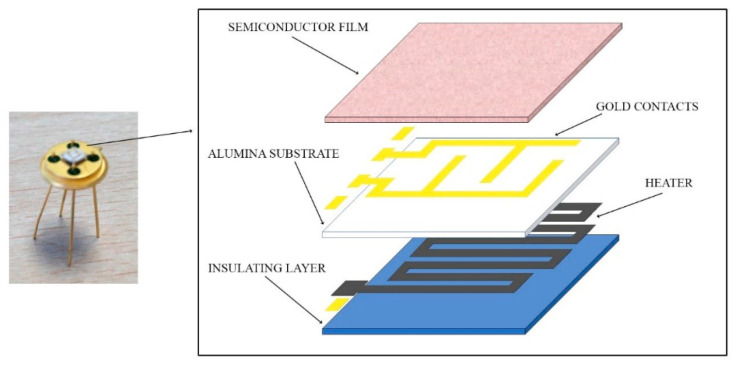
Nanostructured gas sensor bonded with gold wires to its support and scheme of its structure.

Gas sensors are composed of chemoresistive nanostructured semiconductors, where chemoresistivity is the property of a material to vary its electric resistance as a function of the chemical reactions on the surface. Sensors are composed of a thick film (about 200-µm thick) of nanostructured metal-oxide (MOX) materials, synthesized in the SL of UNIFE and then printed using a serigraphic (screen-printing) technique onto an alumina substrate (2.54 mm × 2.54 mm), two comb-shaped gold electrodes, and a platinum heater for heating the sensors to the desired working temperature. The sensors are shown in Figure 2. 

Each sensor produces a voltage output signal dependent on the interaction with the gas or gas mixture present on its surface from which the Response (R) is extrapolated. R is defined as ΔG/G, where ΔG is the difference between film conductance in the presence of the gas (G_gas_) and in environmental air (G). Further information regarding the calculation of R as a function of the type of material can be found in the references [13,14,15,23,24]. 

**Figure 3:**
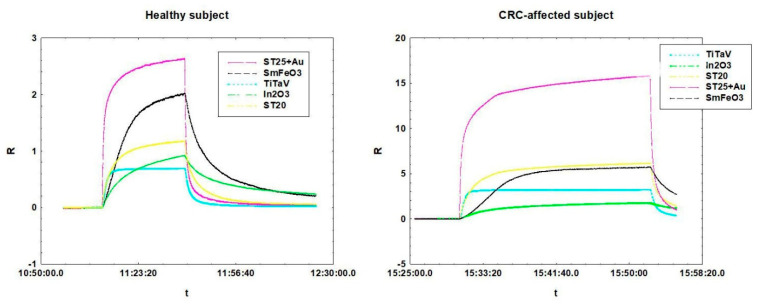
Dynamical response curves of the sensor array employed for the analysis on two positive FIT subjects. On the left graph the subject resulted negative to colonoscopy, on the right graph the subject was CRC affected.

From the trend of the response curve it is possible to deduce crucial features, fundamental to the recognition of the gases analyzed. An example of two sensor dynamic response curves is shown in Figure 3. Though both measurements were made on FIT-positive subjects, in the left graph the patient resulted negative to colonoscopy, while on the right the patient was CRC affected. 

Each single sensor is sensitive to a wide range of compounds and responds uniquely to the gases composing the mixture. Moreover, the response to the total mixture is not the direct sum of each single component contribution, due to complex chemical reactions on the sensor surface. For this specific reason, there are interference contributions between the gases that can conceal information contained in the pure sensor response. By only analyzing the response values, it is not immediately possible to state the difference among the two classes (healthy and tumor-affected); therefore, it is necessary to deepen the analysis with classification algorithms, capable of highlighting such hidden information (see Section 2.3). Sensors selected for the analysis, out of about twenty different materials, are listed in Table 1. 

To perform the analysis, a fecal sample must be inserted into the red sample box of SCENT A1 (Figure 1). By activating the switch on the front side of the device, a stream of humidity-stabilized ambient air conveys the fecal fumes to the sensors, which provide the output response values. A single test takes less than ten minutes, in addition to the time necessary to clean the pneumatic lines from the exhalations. 

After a test, sensor responses are analyzed by means of software developed by the authors, based on the combination of an algorithm for extrapolation of features and support vector machine (SVM) technique for data classification [25,26], which gives the result of the analysis.

The algorithm for data collection of the SCENT A1 consists of specialized software, created in LabVIEW. It allows recording of the output voltage related to each sensor during the analysis, automatically generating their dynamic response curves. The data collected are then analyzed by a second set of software, whose construction and optimization has proceeded hand in hand with the course of the protocol itself. Some of the results of these first phases have already been published in previous works [24,27]. 

The classification software relies on the SVM classifier [25,26], and in the first phase, 20 different parameters, four for each sensor of the array, have been taken onto account: (i) maximum value of the derivative of the response curve (D_max_); (ii) time to reach 10% of D_max_ from the start of the analysis (t_0_); (iii) response (R) at time t; (iv) integral of response curve from t_0_ to t. 

The threshold corresponding to 10% of D_max_ was chosen after extensive analysis, as it emerged as the best compromise between analysis duration and the information collected. Most of the information on the possible positiveness of the sample in relation to the dynamics of surface interaction between sensor and gas is contained within this range.

To make the algorithm more easily adaptable to mass-produced devices, together with the increase of available data, it was necessary to improve the methodology.

In this second phase, to simplify the system, the number of sensors taken into account was reduced to two. After testing all the possible pairs, the sensors chosen were SmFeO3 and ST20 (see Table 1), having proved to be the most discriminating pair. 

A parallel research program is ongoing in order to establish sensors reproducibility, using both fecal samples and common gases in laboratory setups. 

Limiting the number of sensors means dealing with fewer variables that may potentially affect the reproducibility of the system. The method employed is the k-fold cross validation test [26], where the original sample is randomly partitioned into a number (k) of equal sized subsamples. While a single subsample is kept for testing the model (validation data), the remaining k − 1 subsamples are employed in algorithm training (training data). The validation data subsample is then rotated, repeating the cross-validation process k times, ending with a single estimation (positive or negative).

All the authors had access to the study data and have reviewed and approved the final manuscript.

### 2.3. Clinical Validation Protocol

Following approval by the Ethics Committee, a Protocol for the clinical validation of the SCENT A1 device was evaluated from May 2016 to July 2019. The protocol (number: 151298) involved the following partners:

- St. Anna Hospital of Ferrara;
- University of Ferrara—Department of Physics and Earth Sciences;
- AUSL of Ferrara—Department of Public Health;
- SCENT S.r.l.

All participants in the FIT screening program in Ferrara (men and women aged 50–69 years resident or domiciled in Ferrara) who resulted positive were invited to take part in the experimental study of SCENT A1 device before undergoing colonoscopy investigation [28]. The invitation to the protocol was suspended from July to August each year due to the high temperatures and a humidity degree in the environmental air that can affect measurements. This is completely in line with the suspension of the FIT, which decreases specificity in the warmer months [29]. No a priori selection on subjects has been made. All the screening users who resulted FIT positives were invited to the SCENT A1 test before knowing their participation in the gold standard (colonoscopy). Only people who have already had CRC were excluded.

Gas sensor responses are strongly affected by humidity and temperature variations [30,31]. Temperature can also affect sample conservation. This problem can be overcome in countries with strong climatic variations by performing the test under environmentally controlled conditions and by means of a carbon filter at the entrance of the device, as well as paying attention to sample collection and transport. We plan to create polystyrene cases for the transport of containers with feces to limit thermal variations, and to rely on specialized vehicles with refrigerated compartments already in use for the transport of other sensitive biological samples. 

The inclusion and exclusion criteria for the study are summarized in Table 2.

Patients who resulted positive to FIT were invited by the Ferrara Screening Center to undergo the experimental SCENT A1 test before colonoscopy and related gastrointestinal preparation, after obtaining informed consent. 

Once consent had been granted, patients received a standard container (ARTSANA SpA Feces Container STER 18140) for feces collection (to be returned frozen) as well as a leaflet with instructions for correct collection. Information regarding the collection procedure is reported in our previous work [32], in particular focusing on the amount of fecal material needed. Only a small amount is sufficient (about a fifth of the container volume), as too high a quantity may complicate the measurement process. As stated in feasibility studies, the VOC (volatile organic compound) emission flow depends mainly on the exposed surface and not to the quantity of material. Immediately after collection, patients are instructed to store the sample in a domestic freezer. A summary of the general lines of this data acquisition protocol is shown in Table 3.

Sample transportation took place in a thermal bag. The sample should be kept frozen (−18 °C) inside a domestic freezer to within one hour of the SCENT A1 test to inhibit bacterial activity that could alter the measurement. For future tests, we are planning to design polystyrene transport container in order to guarantee a lower thermal variation of the sample, enabling transport even during the hottest months.

Multiple tests using the same sample were avoided to guarantee the perfect conservation of the fecal compound. Furthermore, multiple samples from the same patient were not taken into consideration, in order not to complicate the collection procedure and to align with the FIT protocol in Ferrara. 

In the first year of the Protocol, during the interview with FIT positives, in addition to SCENT A1 tests, a volunteer physician developed a questionnaire for patients addressing issues concerning familiarity, symptoms and other pathologies, used by the doctor to arrive at a personal judgment of the patients’ state of health. A comparison between these observations and colonoscopy outcomes showed that sensitivity and specificity of an expert eye are, respectively, of 11% and 96%, evaluated on a sample of 131 subjects [24]. In this evaluation, all the CRC (starting from low-risk adenoma stage) are included in the category “positives”. However, if only the high-risk adenomas and cancers were considered as positives (with low-risk adenomas in a separate class), the sensitivity would have been null. Despite the data being only qualitative, this information gives an idea of the crucial role of an efficient preventive screening system for CRC. 

### 2.4. Data Analysis

The samples data collected by SCENT A1 were analyzed using the method illustrated above by setting k = 3, chosen after numerous trials, to establish the sensitivity and specificity of the method. In the study protocol, the colonoscopy was chosen as the gold standard with which to compare the SCENT A1 outcomes.

For each CRC-affected patient the following tumor-related parameters were recorded: histological type, lesion size, degree of dysplasia, lesion site, number of lesions removed and recovered, type of surgery, TNM and Dukes stage.

In the organized screening program, data of colonoscopies were divided into three categories, based on their degree of risk:

- POS—e.g., three or more adenomas; one adenoma larger than 10 mm in size, adenoma with villous component or high-grade dysplasia (HDG);
- LOW—e.g., 1–2 adenomas smaller than 10 mm in size; tubular adenoma or low-grade dysplasia (LGD);
- NEG—healthy subjects.

To evaluate the diagnostic accuracy of the SCENT A1 device, the protocol required the construction of a binary variable. Indeed, FIT-positive patients subjected to colonoscopy were grouped into two macro-categories dependent on the gold-standard outcomes:

(1) negative to colonoscopy (healthy subjects);
(2) positive to colonoscopy (all types of colorectal adenomas and carcinomas).

In this manner a binary variable is obtained, which allows the construction of a contingency table, with which to calculate the diagnostic power parameters of SCENT A1 (sensitivity, specificity, accuracy, positive predictive power, negative predictive power).

## 3. Results

Study recruitment lasted from May 2016 to July 2019; 500 subjects adhered to the study, almost half of the total of FIT positives. A demographic analysis of both the participants and non-participants in the SCENT A1 test was performed, in order to demonstrate their average overlap in health status and age. This step is essential for ensuring that the test can be used on anyone, and not just on a population with specific characteristics.

The number of positive FITs in Ferrara between May 2016 and the end of July 2019 amounted to 1130, of which 630 did not undergo a SCENT A1 test for various reasons. Of these, 438 patients who performed the colonoscopy during the summer periods were not invited to the test and 192 refused to take part. These 192 subjects were divided into three categories, according to the reason for refusal: (i) temporary absence of the doctor in charge of the protocol; (ii) not eligible for SCENT A1 (IBD or medication intake); (iii) refusal for personal reasons.

Observing the complete database concerning the 630 non-participants in the SCENT A1 test, from which 68 refusals or documented colonoscopies were subtracted (leaving 562), the following percentages emerged by observing their catalogued colonoscopy results (gold-standard): 74.4% were negative, of a total 418; 25.6% were positive (with any type of lesion/lesions), of a total 144.

The FIT-positive samples subjected to analysis with SCENT A1 totaled 398. The remaining samples (102) were not taken into account, due to collection or conservation errors by the patients or because of the lack of a colonoscopy result (eight rejected colonoscopies, five colonoscopies not performed for health reasons or inadequate preparation). The highest number of conservation errors occurred mainly during the first year of measurements, due to problems in communicating with patients, the study still being in the experimental stage. These errors progressively decreased thanks to the refinement of the instructions given in the leaflet and to the accumulated experience of the doctor in charge.

The gold-standard outcomes are as follows:

(1) Negative on colonoscopy: healthy subjects, 260 in total;
(2) Positive on colonoscopy: all types of colorectal adenomas and carcinomas (54 low-risk adenomas and 84 high-risk adenomas and carcinomas), 138 in total.

**Figure 4:**
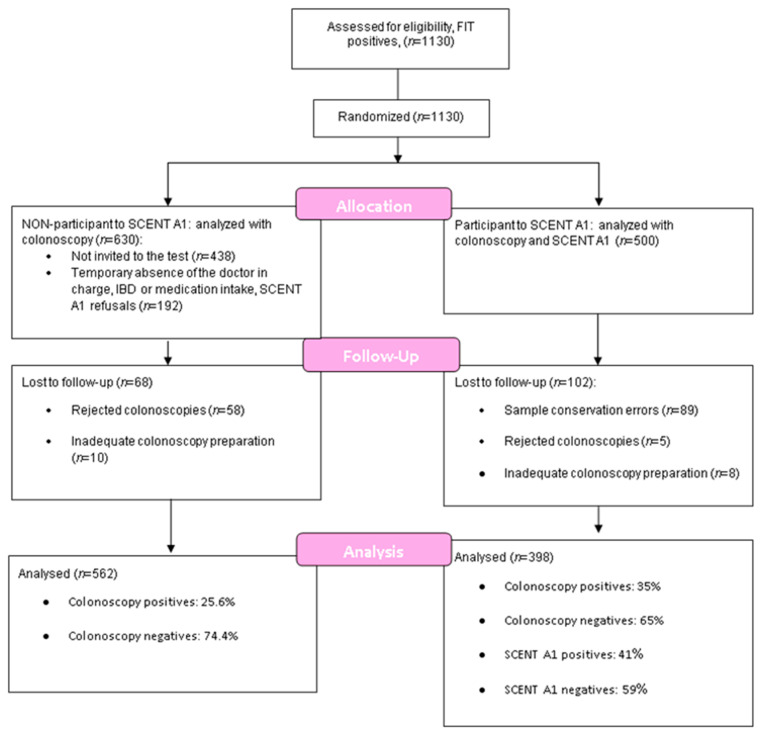
Flow-chart of the study.

Performing the same calculation on the 398 subjects the same calculation as that carried out on the 562 non-participants in the SCENT A1 test, there was a similar percentage of negative and positive subjects: 35% of positive and 65% of negative. This small discrepancy may be due to the fact that in our study we excluded patients with IBD, generally false positives to FIT (positives to FIT because of non-tumoral bleeding and negatives to the gold standard). The two populations resulted comparable based on their health status. Figure 4 shows a flow-diagram summarizing the numbers relating to this study (Consort Flow Diagram).

By cataloguing the total population based on age, the two groups (adherent and non-adherent to SCENT A1) resulted superimposable. A detailed description is given in Table 4.

From these data, it emerged that the two populations resulted comparable and that in both populations the highest percentage of people is aged in the range 65/69+. This could be related to one major factor, namely patient loyalty to the screening program since 2005.

**Figure 5:**
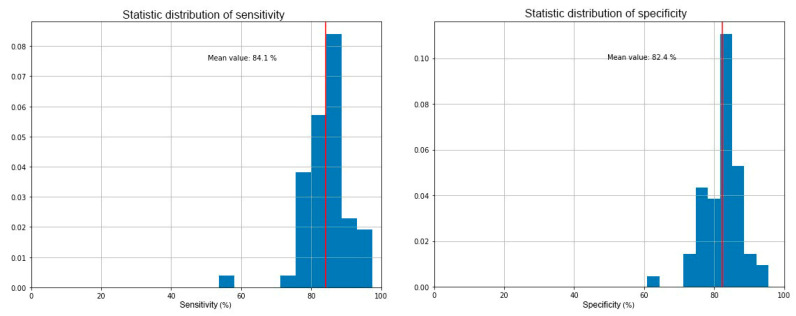
Sensitivity and specificity distributions. The mean values obtained are 84.1% and 82.4%, respectively.

**Figure 6:**
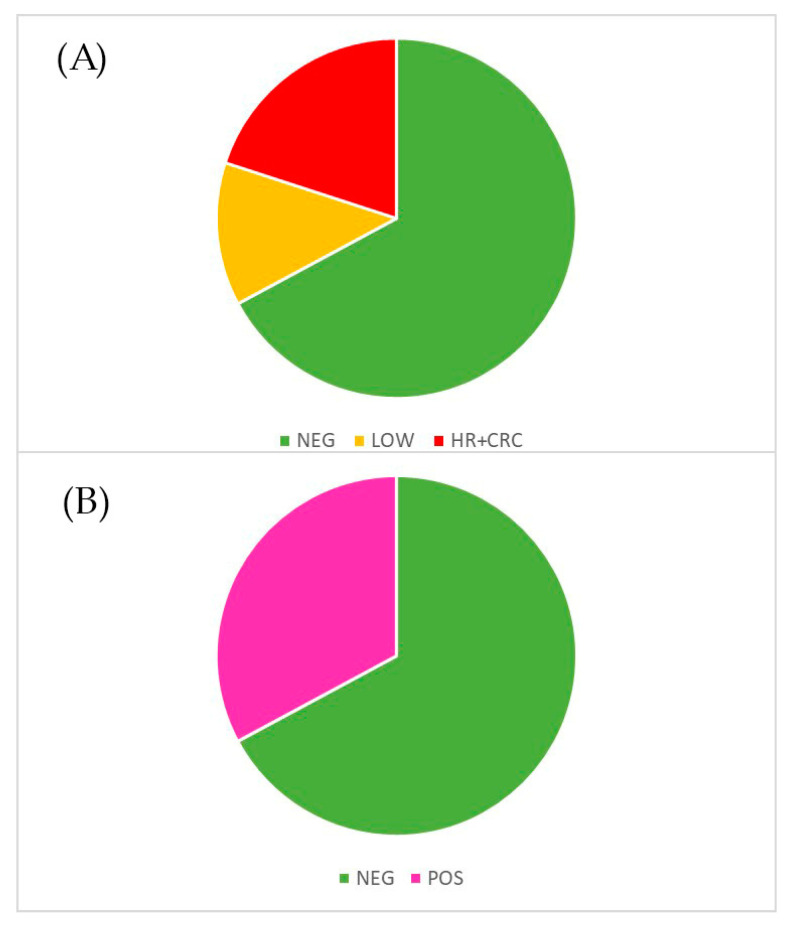
All the data (on 398 FOBT positives) with the two types of division into categories depending on colonoscopy outcomes. (**A**) Division into three categories of risk: NEG (negative to colonoscopy), LOW (low risk adenomas) and CRC+HR (carcinomas and high-risk adenomas); (**B**) division into two categories: NEG and POS (CRC + HR + LOW).

**Figure 7:**
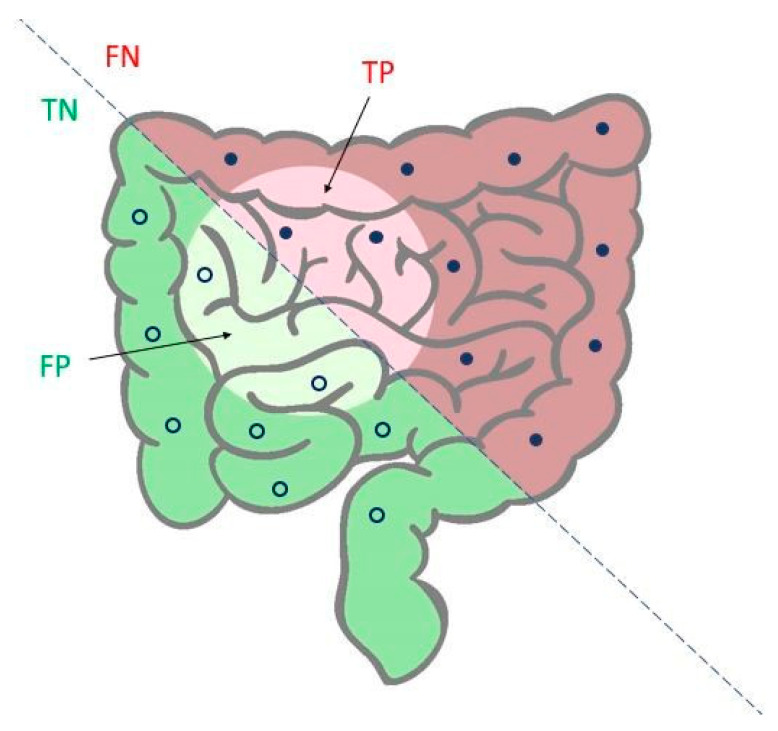
Graphic that represents total data classifications: negatives (TN and FP, empty dots) and positives (TP and FN, filled dots).

In Figure 5, sensitivity and specificity obtained with the SVM-based algorithm are shown, with average values of 84.1% and 82.4%. These parameters are mathematically defined in Table 5, where TP, TN, FP and FN are the abbreviations that respectively indicate true positives, true negatives, false positives and false negatives. Figure 6 shows all the data with the two types of grouping compared, while in Figure 7 an explicative image illustrates graphically the meaning of sensitivity and specificity. 

The contingency table (Table 6) shows the results of the SCENT A1 device in accordance with the results of the gold standard. Based on these calculations, the diagnostic power parameters of SCENT A1 resulted as follows:

- sensitivity = 84% (77–89%)
- specificity = 82% (77–86%)
- accuracy = 83% (79–86%)
- PPV = 72% (64–77%)
- NPV = 91% (86–84%)

In order to guarantee a sufficient statistical power (defined as the probability of a test of being considered as meaningful) the numerosity of the sample was calculated so as to guarantee a statistical power of 95%. The calculation was based on the equation in reference [33], and assuming a 95% confidence interval, the calculation provides 356 samples. An expected prevalence of 25% was chosen, which is the PPV for screening colonoscopies [27], carried out as a second-level assessment (the gold-standard test for this study) and an absolute precision of 4.5%.

## 4. Discussion

The decision to group carcinomas and both high- and low-risk adenomas into a single class rather than two separate classes as before [32], was taken in order to test the accuracy of the device using a binary statistic test. 

Although the degeneration risk over time of low-risk adenomas is still very low and often irrelevant, we preferred to include them within positives to SCENT A1, to ensure greater control over the health status of participants and to increase population consent for this screening method. This grouping action, however, given low-risk tumors are often very similar to healthy tissue with regards to cellular emissions [17], lowers specificity and sensitivity with respect to the division into three categories. As already evident from the preliminary analysis [27], in fact, the major error of classification concerned only low-risk tumors (only 57%), since, being comparable to healthy cells, they emit volatile compounds halfway between healthy and tumoral samples. This study was geared to meet a strong need to improve CRC screening. In Italy and many other countries FIT is adopted by organized screening programs. This test shows a high number of false positives, leading to numerous non-operative colonoscopies with related risks (e.g., bowel perforation) and high hospitalization costs. 

The patented device shown here, SCENT A1, is designed to perform an innovative, non-invasive, in vitro analysis of fecal samples exhalation composition, giving an overview of the health status of the patient. 

The clinical validation of this device, performed in Ferrara (Italy), showed that SCENT A1 was capable of correctly distinguishing between healthy and tumor-affected subjects, thanks to a nanostructured gas sensor array calibrated for this purpose: the sensitivity and specificity obtained by this method resulted as 84.1% and 82.4%, respectively. The PPV result was 72%. This is an extremely encouraging result given that the FIT-PPV with respect to colonoscopy is much lower (25–35%) [27], resulting in a high number of false positives (positives to FIT and negative to colonoscopy), with a great improvement in the efficiency of the screening program. By considering the CRC screening data collected in the Province of Ferrara (Italy), the total number of FIT positives in the period from January 1, 2014, to December 31, 2019, was of 8377. Of these, 7690 colonoscopies have been performed. The total number of lesions was 2530 (148 cancers, 947 high-risk adenomas, 1435 low-risk adenomas). This means that only the 33% of lesions have been correctly identified by FIT (and only less than 2% if only cancers are considered as true positives). The other FIT positives that performed colonoscopy were all false positives, leading to a total of 5160 unnecessary colonoscopies. By employing SCENT A1 on all the FIT positives as a triage test in combination to FIT and colonoscopy, this number will be drastically reduced, thanks to the high specificity (82%) of SCENT A1.

The resulting value of sensitivity is also very encouraging. This is because currently the FIT positives undergo colonoscopy, while the negatives are invited to a FIT two years later. With our method, FIT positives that resulted negative to SCENT do not have to undergo colonoscopy though they are, however, recalled for a FIT after one year not two, to account for a sensitivity value below 100%. Nonetheless, many more people are encouraged to adhere to the screening, knowing they will not necessarily undergo a colonoscopy if they show positive.

The population of participant and non-participants to the three-year protocol resulted to be superimposable basing on their age and health status, confirmed by the gold standard. This guarantees the potential universality of the SCENT A1 test.

This result shows the potentialities of SCENT A1 analysis, if performed on all FIT positives, to eliminate almost two-thirds of non-operative colonoscopies with a significant reduction in screening program costs for the NHS and greater protection for doctors and patients. 

Even the colonoscopy as the gold standard has detection limits, that is, it is not capable of discriminating with absolute certainty between healthy and CRC-affected subjects. Colonoscopy quality has varied in reports from different endoscopists. For this reason, over the last decade, a series of quality indicators for colonoscopy have been described. Currently, the main quality indicator among endoscopists is the adenoma detection rate (ADR). This will need to be taken into account in the analysis of the diagnostic power of SCENT A1.

As anticipated, a double check in the preventive screening phase will also serve to promote greater awareness and adherence to screening by the population, so far reluctant to take part for fear of colonoscopy. In fact, only a little over 50% of interested parties currently adhere [8]. The efficacy of CRC screening is determined by the degree of participation and the diagnostic yield of the test. Use of the FIT has increased participation rates, because it is user friendly, a single sample suffices, and no dietary restrictions are imposed prior to the test. Because the FIT is more sensitive than the G-FOBT, the number of false positives and the demand for invasive tests has increased. Consequently, the cut-off value of the test must be adapted to each region, taking into account the availability of endoscopic resources. The FIT also exhibited superior detection of advanced adenomas compared to the G-FOBT. This feature promotes treatment in early stages and prevents the formation of cancer. Another problem to consider is the low acceptance of colonoscopy in some countries. At present, the only technical limitation to the instrument in its prototypal form is the analysis time, lasting about 30 min, in order to completely clean the pneumatic lines after a measurement. In future, improvements will be made to speed up this procedure, allowing the instrument to carry out as many analyses as possible in the shortest time.

## 5. Conclusions

The SCENT A1 test is an effective, economic and easy to use method to improve the current screening efficiency, as it relies on a collection procedure similar to FOBT, the difference being that the sample must be stored in a domestic freezer. A multicenter protocol will be started, by employing two further productions of the SCENT A1 device. The future protocol will have the aim both of confirming the accuracy of the method and of analyzing fecal samples of FOBT-negative patients. 

Furthermore, the Department of Public Health has already invited patients who resulted negative to colonoscopy in the recently concluded protocol to resubmit a FIT after two years. This step will be fundamental in verifying the accuracy of the colonoscopy itself, if a tumor lesion is present. Since these subjects have already been analyzed with SCENT A1, the double comparison with FIT and colonoscopy will serve to evaluate the possible capability of our device in identifying interval cancers [34]. Moreover, if employed by the NHS of countries where screening systems are not currently in place, this method will significantly reduce the mortality rate related to CRC.

## Figures and Tables

**Table 1:** List of sensors chosen after a feasibility study for the most performing array for SCENT A1. These sensors have been employed in the clinical validation protocol. The sensor names refer to an internal code and are not the chemical compound’s name.

Name	Working T (°C)	Materials
SmFeO3	350	Iron and Samarium oxides
TiTaV	450	Titanium, tantalum and vanadium oxides
ST20	450	Tin and Titanium oxide (20%)
In2O3	350	Indium Oxide
ST25 + Au	450	Tin and Titanium oxide (25%) with the addition of gold nanoparticles

**Table 2:** Inclusion and exclusion criteria of the clinical validation protocol patients.

Inclusion Criteria	Exclusion Criteria
patients (both male and female) between 50-69 years with positive FIT, adherent to second level screening (colonoscopy or colon TAC).	patients with known diagnosis of IBD; patients who took antibiotics in the previous month; patients who recently (in the previous month) took probiotics and laxatives.

**Table 3:** Summary of data and sample collection before SCENT A1 test (and then colonoscopy).

Preliminary Interview	Fecal Samples
informed consent;	it must be collected before colonoscopy preparation;
kit delivery;	it must be conserved not more than one night in freezer and, after that, delivered directly to Centro Screening;
anamnesis registration of a brief survey to exclude interfering conditions for the VOCs analysis.	sigillated and conserved in freezer before other analyses.

**Table 4:** Population cataloged by age.

Population	Age 50/54	Age 55/59	Age 60/64	Age 65/69+	Total
**NO SCENT A1 (#)**	130	114	132	186	562
**SCENT A1 (#)**	93	91	79	135	398
**NO SCENT A1 (%)**	23	20	24	33	100
**SCENT A1 (%)**	23	23	20	34	100

**Table 5:** Mathematical definitions of sensitivity, specificity, precision, NPV and accuracy. TP, TN, FP and FN are the abbreviations that indicate respectively true positives, true negatives, false positives and false negatives. P and N are positives and negatives.

Quantity	Definition
Sensitivity (TPR: true positive rate)	TP/P = TP/(FN + TP)
Specificity (TNR: true negative rate)	TN/N = TN/(FP + TN)
Precision (PPV: positive predictive value)	TP/(TP + FP)
NPV: negative predictive value	TN/(TN + FN)
Accuracy	TP + TN/(P + N)

**Table 6:** Contingency table that shows the results of SCENT A1 test compared with the gold standard.

		GOLD STANDARD (Colonoscopy)		
		Positive	Negative	Total
**Test to be validated (SCENT A1)**	positive	116	46	162
	negative	22	214	236
	total	138	260	398
